# CT-Based Local Distribution Metric Improves Characterization of COPD

**DOI:** 10.1038/s41598-017-02871-1

**Published:** 2017-06-07

**Authors:** Benjamin A. Hoff, Esther Pompe, Stefanie Galbán, Dirkje S. Postma, Jan-Willem J. Lammers, Nick H. T. ten Hacken, Leo Koenderman, Timothy D. Johnson, Stijn E. Verleden, Pim A. de Jong, Firdaus A. A. Mohamed Hoesein, Maarten van den Berge, Brian D. Ross, Craig J. Galbán

**Affiliations:** 1 Department of Radiology, University of Michigan, Center for Molecular Imaging, Ann Arbor, MI United States; 2 Department of Respiratory Medicine, University Medical Center Utrecht, Utrecht, The Netherlands; 3 University of Groningen, University Medical Center Groningen, Department of Pulmonary Disease, Utrecht, The Netherlands; 4 Department of Biostatistics, University of Michigan, Ann Arbor, MI United States; 5 Lung transplant Unit, Department of clinical and experimental medicine, KU Leuven, Leuven Belgium; 6 Department of Radiology, University Medical Center Utrecht, Utrecht, The Netherlands

## Abstract

Parametric response mapping (PRM) of paired CT lung images has been shown to improve the phenotyping of COPD by allowing for the visualization and quantification of non-emphysematous air trapping component, referred to as functional small airways disease (fSAD). Although promising, large variability in the standard method for analyzing PRM^fSAD^ has been observed. We postulate that representing the 3D PRM^fSAD^ data as a single scalar quantity (relative volume of PRM^fSAD^) oversimplifies the original 3D data, limiting its potential to detect the subtle progression of COPD as well as varying subtypes. In this study, we propose a new approach to analyze PRM. Based on topological techniques, we generate 3D maps of local topological features from 3D PRM^fSAD^ classification maps. We found that the surface area of fSAD (S^fSAD^) was the most robust and significant independent indicator of clinically meaningful measures of COPD. We also confirmed by micro-CT of human lung specimens that structural differences are associated with unique S^fSAD^ patterns, and demonstrated longitudinal feature alterations occurred with worsening pulmonary function independent of an increase in disease extent. These findings suggest that our technique captures additional COPD characteristics, which may provide important opportunities for improved diagnosis of COPD patients.

## Introduction

Chronic obstructive pulmonary disease (COPD) is a major cause of morbidity, mortality, and healthcare cost worldwide with an estimated global prevalence of approximately 12% of adults aged ≥30 years in 2010 and rising with the aging population^1, 2^. Recent reports found that COPD etiology varies among populations, including risk factors such as tobacco smoke, cooking fuels, environmental pollution and family genetics^2^. This has led to the current understanding that COPD covers a wide spectrum of pathophysiologies^3, 4^. Clinical presentation and monitoring of COPD have been described primarily through spirometry as pulmonary function measurements. Although highly reproducible, these measures, such as forced expiratory volume in one second (FEV1), assess the lungs as a whole and are unable to differentiate two key components of COPD: emphysema and small airways disease. In addition, spirometry does not provide spatial context for regional heterogeneity of these components. X-ray computed tomography (CT) has addressed some of these limitations by allowing clinicians to verify emphysema in patients exhibiting loss of pulmonary function. Even with these techniques, COPD is often undiagnosed in early stages, impeding proper treatment with the disease progressing to permanent lung damage (i.e. emphysema). Although COPD phenotyping has been prolifically reported in the literature^5–7^, lack of accurate diagnostic tools that identify these unique COPD subtypes have hampered the development of effective therapies. Nevertheless, significant advances in technologies are providing physicians opportunities to shift towards “precision medicine”.

Various strategies have been undertaken to identify metrics that more accurately assess COPD subtypes, such as genetic, molecular and cellular markers as well as medical imaging devices and methodologies. Although advances in biological phenotyping have shown promise in identifying disease heterogeneity in patients^4, 8^, these approaches are generally either global measures or highly invasive. In contrast, medical imaging provides clinicians with a relatively non-invasive and reproducible approach that provides functional information that is spatially defined. Although various instruments (e.g. PET, SPECT and MRI) are heavily investigated as surrogates of pulmonary function and clinical outcome^9^, CT, with its high resolution and lung contrast, continues to be the most widely used medical imaging device in the clinic. As such, advances in this technology would have an immediate impact on patient care.

CT is inherently a quantitative map, where x-ray attenuation is linearly proportional to lung tissue density^10, 11^. Extensive research in CT image post-processing has generated an array of potentially diagnostic and prognostic measures. Filter-based techniques and airway wall measurements have been extensively explored^12–14^. Not only have these methodologies advanced our understanding of COPD, they are also becoming more prevalent in clinic decision-making. In fact, the quantification of discrete phenotypes of emphysema using CT has had an impact on patient care. At present three emphysema patterns (i.e., centrilobular, panlobular, and paraseptal emphysema) have been identified, each of which are strongly associated with a range of respiratory physiologies and functional measures^15, 16^. The understanding that unique spatial patterns of emphysema serve as indicators of COPD subtypes has spawned progress in lobe segmentation algorithms^17, 18^ as well as the need to evaluate CT-based features^19^. Although our understanding related to the clinical implications of the spatial patterns of emphysema is emerging^20^, little is understood about the non-emphysematous component of COPD, commonly associated with small airways disease.

Small airway disease, a major component of COPD, is generally characterized by the presence of inflammation, fibrosis, and mucous plugging, all of which contribute to airflow obstruction. At less than 2 mm in diameter, these airways are essentially invisible to clinical imaging scanners, hindering accurate COPD phenotyping by CT especially when emphysema is radiographically identified. The Parametric Response Mapping (PRM) technique^21^ addressed this limitation. Through the spatial alignment of paired inspiration and expiration CT scans, PRM of CT data delineates and quantifies non-emphysematous air trapping, an indirect measure of small airways disease (SAD), even in the presence of emphysema^21, 22^. The extent of fSAD, as measured by PRM as the relative volume (%PRM) in the lungs, has been reported to be an independent indicator of pulmonary function decline as well as other clinically relevant measures, re-affirming previous histological studies^22–24^. In addition, McDonough and colleagues have shown pathologically in human core lung specimens imaged by micro-CT that small airways disease may in fact serve as a precursor to emphysema^25^. This highlights the potential importance of the independent and non-invasive evaluation of fSAD through PRM^26^. Although the spatial information of fSAD is retained as a 3D PRM classification binary map, studies have primarily focused on the use of a whole-lung measure of fSAD, presented as a relative lung volume, which serves as the extent of this COPD component within the patient. As observed with emphysema, the spatial distribution of fSAD may aid treating physicians by providing them unique diagnostics that serve as a surrogate of clinically meaningful outcomes.

**Figure 1:**
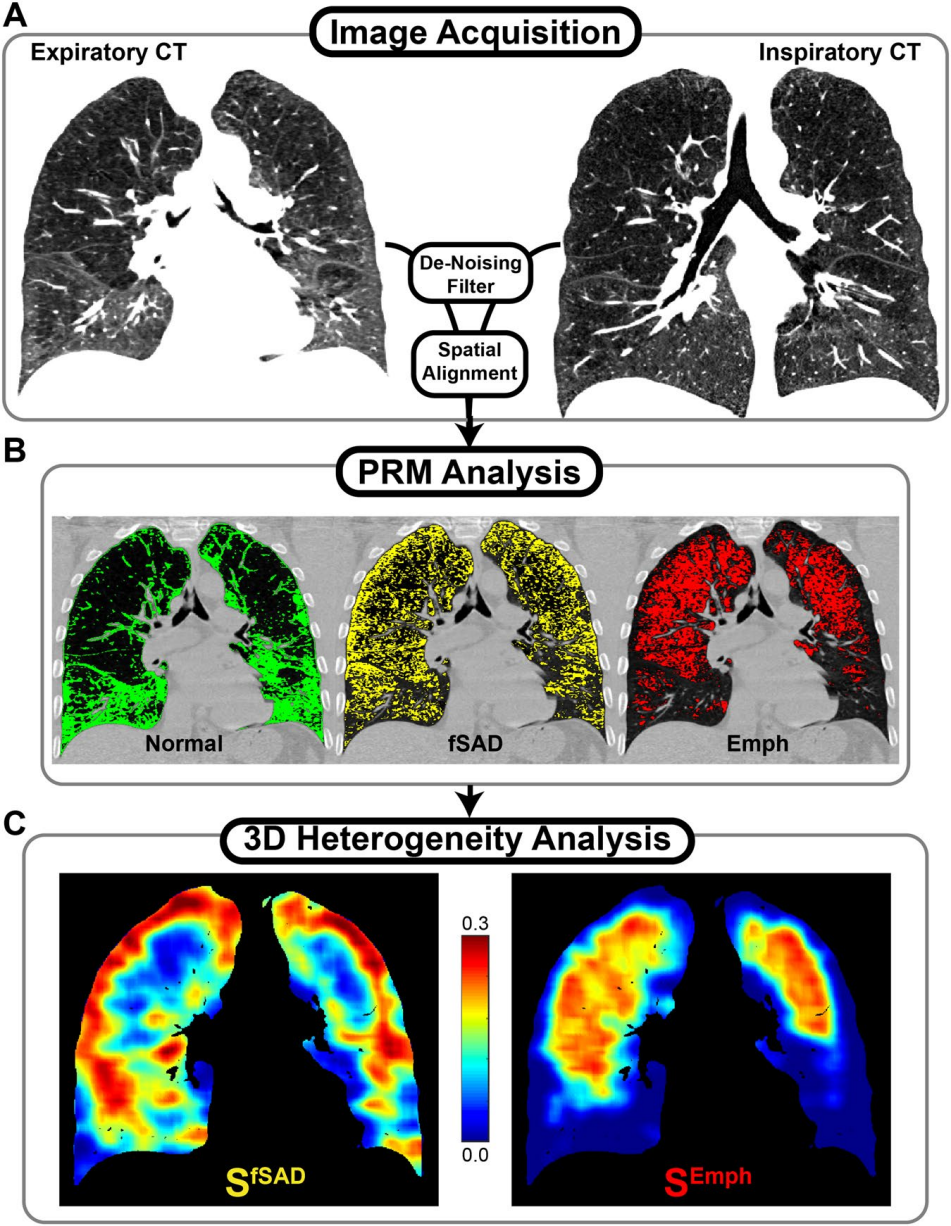
A schematic of the workflow is displayed for generating PRM topological maps. (**A**) CT images are acquired at expiration and inspiration. (**B**) PRM analysis is performed by first segmenting the lungs from the thoracic cavity. Then the CT images are filtered and spatially aligned to the expiration geometric frame. Individual voxels are then classified as normal (PRM^Norm^, green), functional small airways disease (PRM^fSAD^, yellow), or emphysema (PRM^Emph^, red). (**C**) Topological feature extraction is performed on each PRM classification binary map to determine topology metrics. Presented are surface area (S) maps for PRM^fSAD^ (left) and PRM^Emph^ (right).

The present study demonstrates an extension of the PRM approach that extracts local topological features from PRM-derived disease classifications maps (Fig. 1). Using CT scans from COPD patients assessed as GOLD stage 1–4 accrued as part of a well-controlled multi-center clinical trial^27^, we found that disease pattern (i.e. topological features) is correlated with COPD severity independent of disease extent (i.e. relative volume). Through micro-CT analysis of explanted lung cores from a lung transplant recipient with bronchiolitis obliterans syndrome, an obstructive lung disease‚ and longitudinal CT data acquired from a COPD subject, we provide anecdotal evidence that PRM-derived topological features are associated with structural differences and may also reveal trends in progressing obstructive disease. This work demonstrates for the first time that spatial features extracted from PRM^fSAD^ maps, specifically the surface area (S^fSAD^), provide independent predictors of clinical outcome measures, as well as provide illustrative examples that these features are associated with unique airway and parenchyma structures and disease progression.

## Results

### Topological Features

The topological features of the PRM classification binary maps of fSAD and emphysema, defined throughout as PRM^fSAD^ and PRM^Emph^, were determined using the Minkowski Functionals: surface area (S^i^), mean curvature length (B^i^), the Euler-Poincare characteristic (χ^i^), and a condensed descriptor of clustering (α^i^), where i is an index for fSAD or Emph determined by PRM. These measures were determined locally, referred to as “Local”, over sub-volumes of the lung using a moving window approach resulting in a 3D parameter map for each metric, or globally, referred to as “Global”, over the entire lung volume resulting in a single parameter scalar quantity for each metric. For statistical analysis Local values represent the full lung mean value. Four Local and four Global topological metrics were generated from each binary PRM classification map. We first sought to determine the robustness of each parameter by performing a linear regression of the mean of Local parameters to their Global parameters when applied to PRM^fSAD^, all voxels classified as fSAD by PRM, and PRM^Emph^, all voxels classified as emphysema by PRM, binary maps. We observed for the surface area (S) of PRM^fSAD^ and PRM^Emph^ near perfect agreement between Local and Global calculations (Supplemental Fig. 3). Linear regression of Local and Global S^i^ generated R^2^ of >0.999. Increasing complexity of the topology metric was found to demonstrate less correlation between Local and Global calculations with α demonstrating clear disagreement between measures (R^2^ < 0.3; Supplemental Fig. 3). Based on these results, the focus of this study will be on the Local surface area (Local S^i^) as it was found to be robust while retaining spatial information throughout the lungs. This topology metric provides an indication of the number of adjacent like-neighbors within a local volume. A local volume with sparsely distributed like-neighbors (dispersed) would result in an elevated S, while co-localization of like-neighbors (cluster) would result in diminished S for the same relative volume. It must also be considered that, at high and low extremes of relative volume, the possible range in S would intrinsically be reduced by limitations in the number of possible interactions between like-neighbors, which is why topological features presented in this study are always considered in the context of their respective relative volumes. More detailed descriptions of all Minkowski Functionals are provided within the supplement. For completeness, analyses of Local and Global B^i^, χ^i^ and α^i^, as well as Global S^i^, are presented in the supplement.

### Patterns of Disease

**Figure 2:**
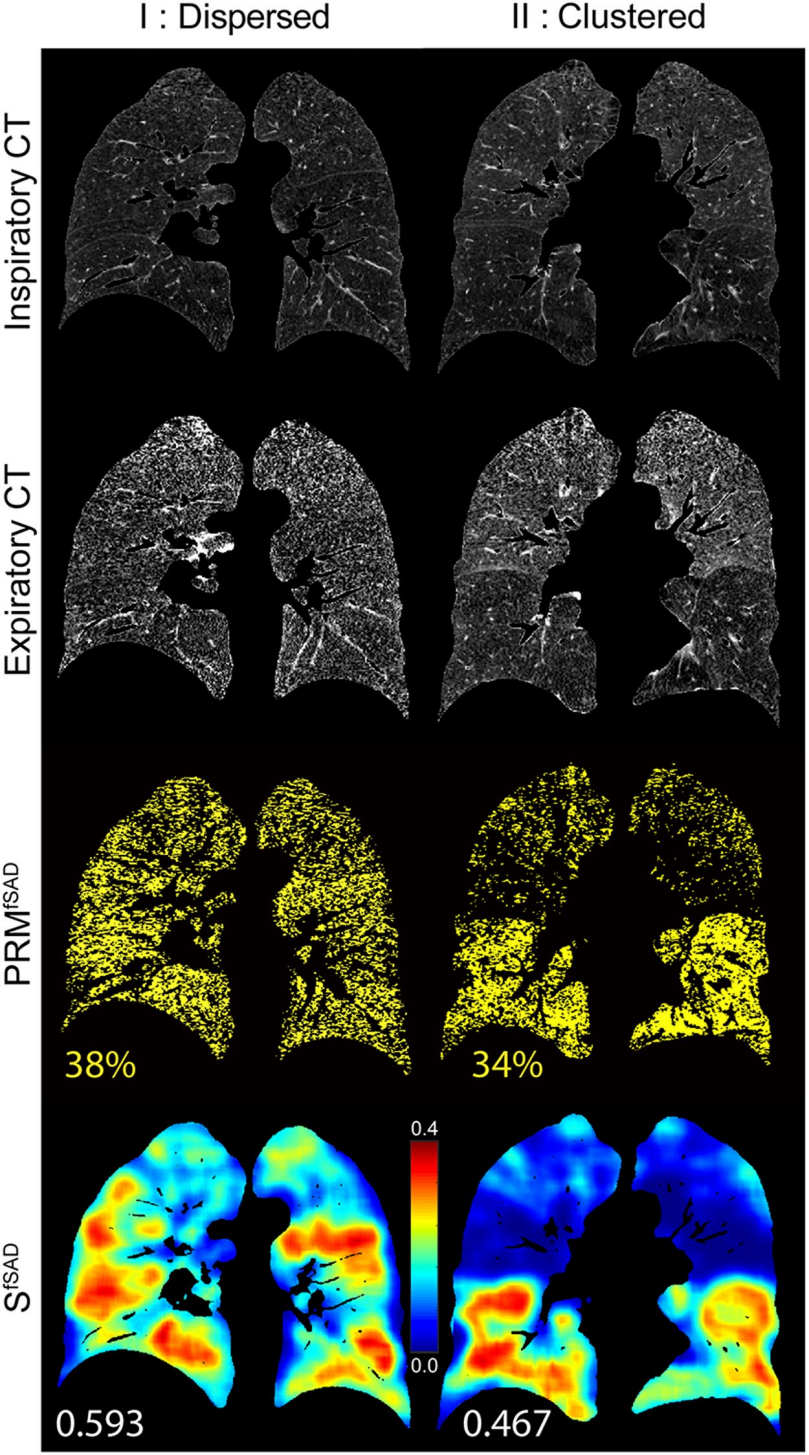
Two cases are presented with varying topological PRM features. Both cases have near-identical spirometry readouts and PRM^fSAD^ relative volumes yet display differing fSAD topological features: dispersed disease (I, left column) and clustered disease (II, right column). A representative slice is shown for each case for the respective (top to bottom) inspiratory CT, expiratory CT, PRM^fSAD^ map, and Local S^fSAD^ map (multiplied by Local V^fSAD^ in order to emphasize regions of substantial disease). PRM^fSAD^ relative volumes and Local S^fSAD^ values are displayed for comparison.

Recent studies have demonstrated a strong correlation of total lung relative volume of PRM^fSAD^ to relevant clinical measures^21, 22^. Nevertheless, the relative volume of PRM^fSAD^, extent of fSAD, has shown wide variability in recent studies resulting in attenuation of its sensitivity to clinical measures. As COPD is a progressive disease, the severity of SAD will vary over time which may explain the large variability observed in the relative volume of PRM^fSAD^ from cross-sectional studies. The strength of our approach is the ability to delineate disease pattern that resides within our PRM classification maps, allowing for further phenotyping of individual patients. We postulate that the topological pattern (i.e. feature) of PRM^fSAD^ may be a correlative contributing factor to the observed variability in the relative volume of PRM^fSAD^ as it relates to clinically meaningful metrics and thus may in fact be diagnostically important. Here we provide two GOLD 2 cases to illustrate unique spatial patterns, i.e. Local S^i^, with similar relative volumes in PRM^fSAD^ (Fig. 2): Subject I (Male, age 73) with dispersed PRM^fSAD^ and Subject II (Male, age 59) with clustered PRM^fSAD^ distributions. Pulmonary function measurements were near identical with FEV1% predicted and FEV1/FVC values of 51.4% and 0.487 for Subject I and 50.1% and 0.497 for Subject II. Both subjects had substantial PRM^fSAD^ (I: 38% and II: 34%; Fig. 2) with negligible PRM^Emph^ (I: 1.4% and II: 1.6%). Local S^fSAD^ maps for these two subjects (Fig. 2) revealed differing patterns within the lungs with mean local values of 0.593 and 0.467 for Subject I and II, respectively. Additional metrics also showed differences in the PRM^fSAD^ topology between Subject I and Subject II (Supplemental Fig. 4). Through our approach we were able to extract pattern information that was represented as an easily interpreted scalar quantity with dispersed disease demonstrating higher values in Local S^fSAD^. We next evaluated our parameters using well-controlled CT and clinical data to determine Local S^i^ as independent indicators of clinically meaningful metrics even when considering PRM relative volumes as measures of extent of fSAD and emphysema.

### GOLD Comparison

**Figure 3:**
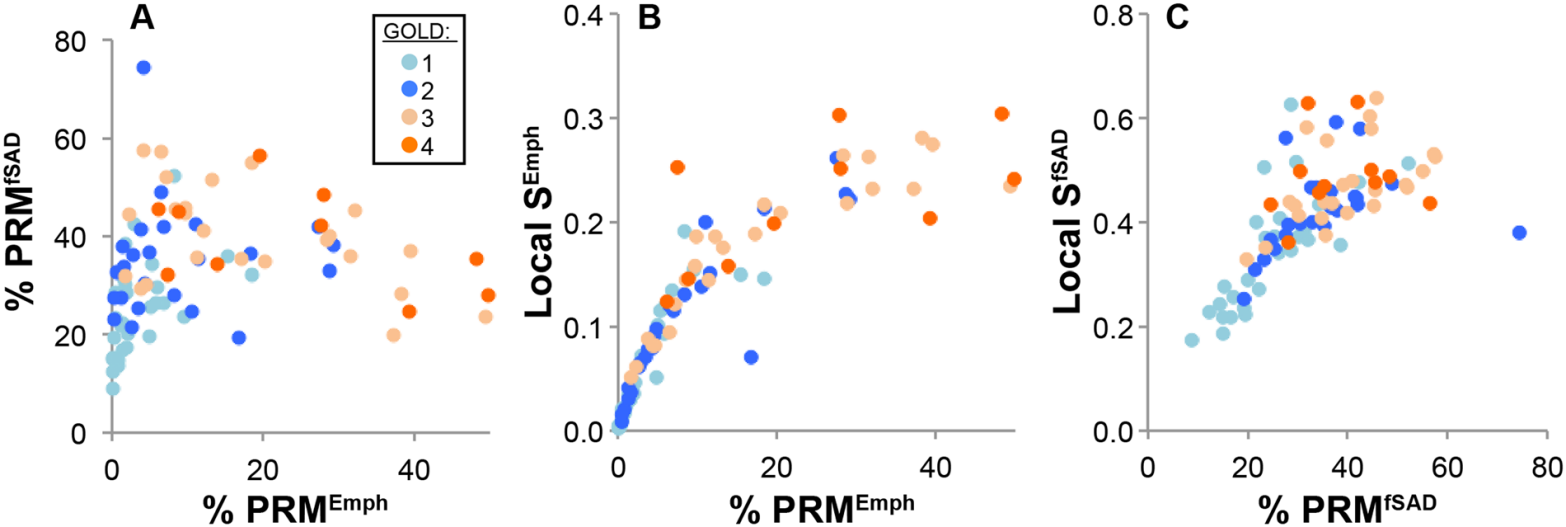
Observable trends between PRM and topological PRM features. Scatter plots are presented for (**A**) %PRM^Emph^ vs. %PRM^fSAD^, (**B**) %PRM^Emph^ vs. Local S^Emph^ and (**C**) %PRM^fSAD^ vs. Local S^fSAD^. Markers are color-coded for GOLD 1 through 4 (see legend). Comparison between %PRM^Emph^ and %PRM^fSAD^ showed trends with increasing disease severity that has been observed in previously published work^21, 22^. Local S^i^ values and respective %PRM^i^ values reveals a strong correlation at low volume fractions showing greater spread with increasing volume fractions.

We evaluated the relationship between Local S^i^ and relative volume of PRM^i^ (Fig. 3) as well as GOLD status over the entire subject population (Fig. 3 and Supplemental Figs 5 and 6). Negligible differences were observed in age, BMI and pack-years between GOLD groups (Supplemental Table 1). The relationship between the relative volumes of PRM^Emph^ and PRM^fSAD^ with increasing GOLD status was consistent with previous reports using COPDGene and SPIROMICS trial data^21, 22^ (Fig. 3A, Supplemental Table 1). For a relative volume of PRM^Emph^ < 10%, a near-linear trend was observed with Local S^Emph^ (Fig. 3B). Subjects with severe to very severe COPD showed a diminished relationship between Local S^Emph^ and relative volume of PRM^Emph^, suggesting increased variability in the emphysema pattern. In contrast to Local S^Emph^, Local S^fSAD^ demonstrated extensive variability for a given relative volume of PRM^fSAD^, even within GOLD status (Fig. 3C). This suggests wide variability in fSAD pattern within this study cohort. Similar results were observed for Global S^fSAD^ measurements (Supplemental Fig. 5). Each of the Local topological metrics exhibited unique trends with increasing GOLD status (Supplemental Fig. 6 and Supplemental Table 2). Nevertheless, the observed variation within GOLD status for our feature patterns may be indicative of COPD subtypes independent of the extent of disease (i.e. relative volume of PRM^fSAD^).

### Clinical Outcomes

Based on our regression analysis, Local S^fSAD^ was found to be a significant model parameter of all clinical outcomes, independently of the relative volume of PRM^fSAD^ (Table 1). Similarly, Local S^Emph^ was a significant parameter for all clinical metrics except for the St. George’s Respiratory Questionnaire (SGRQ) total score and 6-minute walking distance, where the relative volume of PRM^Emph^ was the dominant parameter in the regression models (Table 1). As expected, Global S^i^ generated near identical results to the Local S^i^ (Supplemental Table 3). The remaining Local metrics were found to be weak parameters with little to no contribution to many of the regression models (Supplemental Table 3). This trend substantially changed when the entire PRM^fSAD^ classification map, i.e. Global assessment, was used to calculate a single scalar quantity of χ and α but only when applied to the PRM^fSAD^ classification map (Supplemental Table 3). This discrepancy between Local and Global calculations of the more complex topology was most likely attributed to the scope of data being analyzed, with the small window size not able to capture the same features as the global analysis. Nevertheless, we have demonstrated for the first time that the pattern of PRM^fSAD^, using our technique for extraction of spatial topology, is strongly correlated with clinical readouts even when considering the overall extent of the disease (i.e. relative volume of PRM^i^).

**Table 1:** Multivariate regression results.

		% PRM^i^	Local S^i^
**fSAD**	FEV_1_ (% predicted)	—	<0.0001^d^
	FEV_1_/FVC	0.0114	0.0131^d^
	SGRQ total score	—	0.0006
	6-min walk distance	0.0091	<0.0001
	MMRC dyspnea scale score^a^	—	<0.0001
	BODE score^a^	—	<0.0001
**Emph**	FEV_1_ (% predicted)	—	<0.0001^b^
	FEV_1_/FVC	—	<0.0001
	SGRQ total score	<0.0001	—^d^
	6-min walk distance	<0.0001	—^c,d^
	MMRC dyspnea scale score^a^	—	<0.0001
	BODE score^a^	—	<0.0001

Note: Presented are the *P values* generated from stepwise regression models including the topological index S, respective PRM relative volume, age, gender, and body mass index (BMI). FEV_1_ = forced expiratory volume in one second (% predicted); FVC = forced vital capacity; SGRQ = St. George’s Respiratory Questionnaire; MMRC = Modified Medical Research Council; BODE = body mass index, degree of airflow obstruction, dyspnea, and exercise capacity. ^a^denotes use of a logistic instead of linear regression, ^b^denotes age was significant, ^c^denotes gender was significant, and ^d^denotes BMI was significant. Parameters not included in the model due to lack of significant effects are marked with a dash.

### Case Study 1: MicroCT Analysis of Tissue Explant in BOS

**Figure 4:**
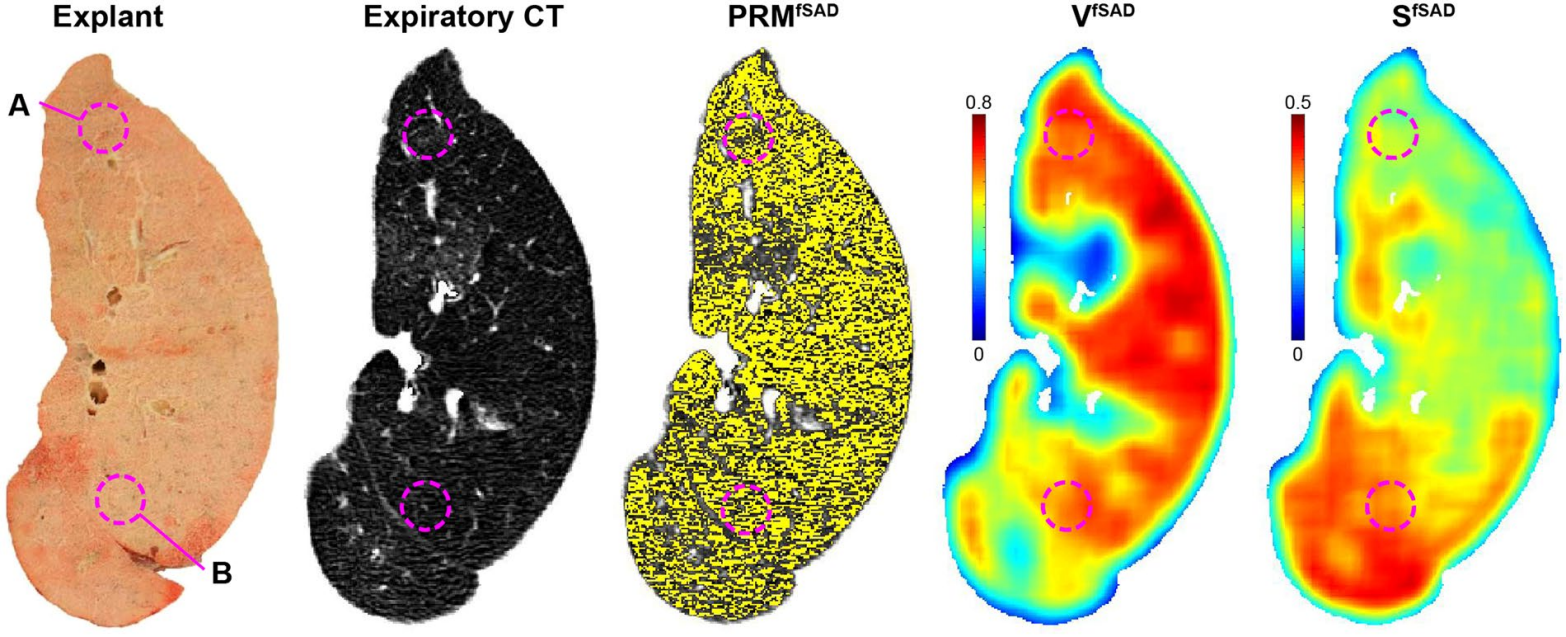
Confirmation of topological features altered by tissue microenvironment. An explanted lung from a single subject diagnosed with bronchiolitis obliterans syndrome was analyzed by microCT. Presented are the explanted lung section, expiratory CT scan, PRM^fSAD^, Local V^fSAD^ and Local S^fSAD^. All CT derived images were spatially aligned to the lung section, allowing identification of core regions (**A**,**B**) on PRM and topological maps.

Here we provide a case that demonstrates our topological metrics reflect the microenvironment of lung tissue with bronchiolitis obliterans syndrome (BOS) as determined by microCT. BOS, a chronic lung allograft dysfunction in lung transplant recipients, is characterized by a spirometric decline, obliterative bronchiolitis (OB) on histopathologic examination and air trapping and mosaic attenuation on CT. The following case is from a 64-year-old male diagnosed with BOS 6 months subsequent to initial transplantation for COPD. Subject underwent re-transplantation 2.5 years later for end-stage BOS. Prior to the later resection of the lung, PRM analysis showed elevated PRM^fSAD^ (56% of the lung volume) in the BOS lung. Although a high volume fraction of fSAD was observed in the analyzed explant section, topological analysis of PRM^fSAD^ revealed varying feature types in different regions that could not easily be identified on the PRM map or CT images alone (Fig. 4). Cored samples (A and B in Fig. 4) were analyzed from two regions of the lung section and found to have varying values in Local S^fSAD^ and χ^fSAD^ (Supplemental Table 4). Observable differences in microenvironment were ascertained by microCT of two core samples from the same explanted lung section. Core A was found to have more severe disease with two obliterative bronchioles, whereas no obliteration was observed in Core B. These results confirm that the Local S^fSAD^ and χ^fSAD^ may be sensitive to the degree of obliteration, and possibly severity of small airway disease.

### Case Study 2: Monitoring 5 Year Progression of COPD

**Figure 5:**
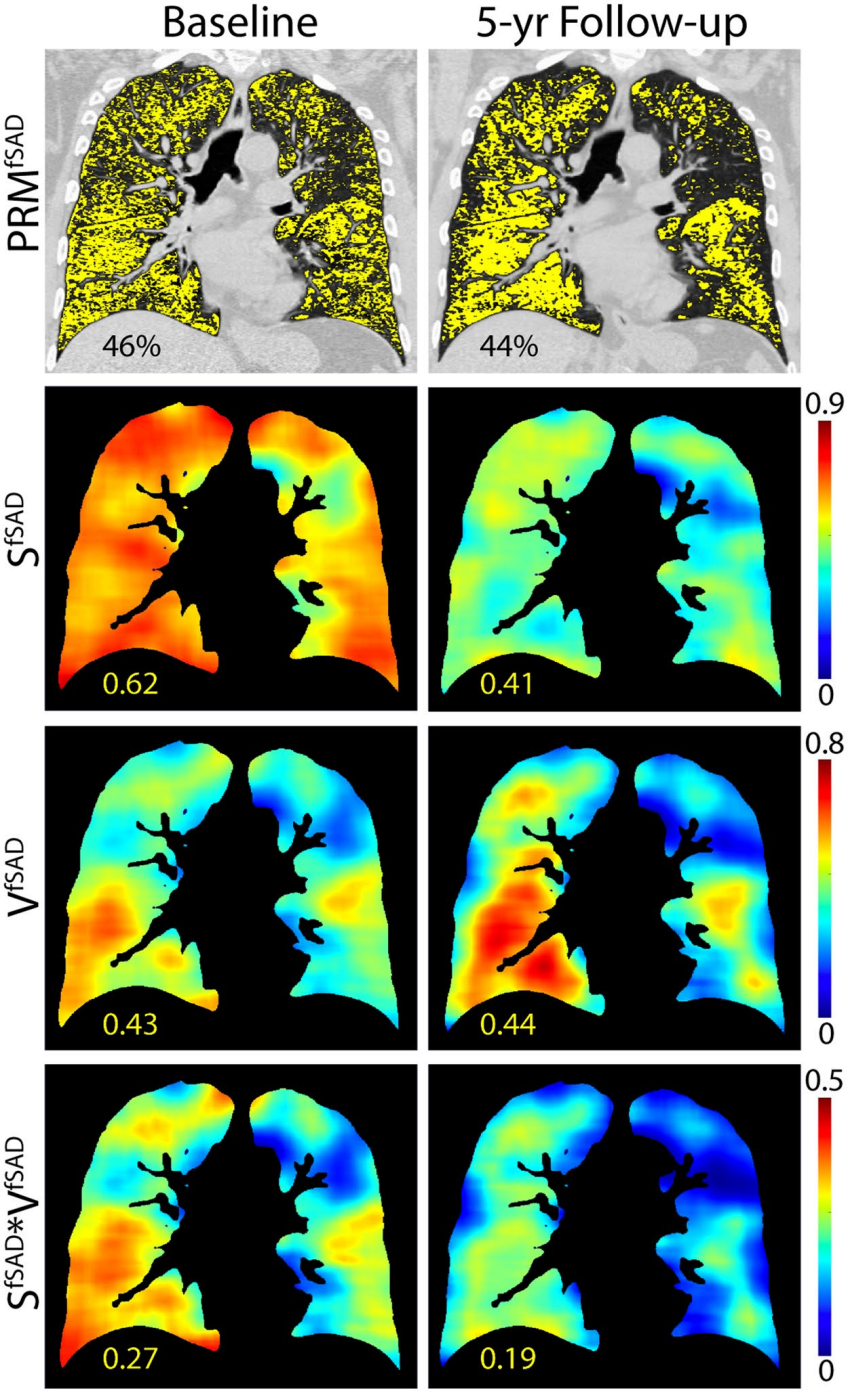
A subject with COPD staged at baseline with GOLD-2 (first column) with 5-year follow-up (second column) revealing a progression to GOLD-3. No substantial change in %PRM^fSAD^ was detected (first row), however a striking drop in Local S^fSAD^ was found (second row). This demonstrates a potential pattern of progression, with diffuse disease coalescing into more focal distribution.

Next we provide a longitudinal case demonstrating the potential of our PRM topological feature technique to capture disease progression as assessed by spirometry with negligible changes in the relative volume of PRM^fSAD^. The subject was a 66-year-old male accrued as part of the COPDGene 5-year trial. Upon enrollment, this subject was diagnosed with GOLD 2 COPD (FEV1% predicted of 56.1%). At the 5-year follow-up, the subject demonstrated spirometric decline (FEV1% predicted of 40.6%) subsequently diagnosed with GOLD 3 COPD. No substantial increase in the relative volume of fSAD as measured by PRM was observed, yet the Local S^fSAD^ was found to decrease over the 5-year period (0.62 to 0.41) suggesting clustering of disease (Fig. 5). Here we demonstrate that our PRM topological metric is sensitive to progressive disease that manifests as a local coalescing of obstruction brought about by small airway disease.

## Discussion

We have introduced a unique CT-based methodology for classifying COPD phenotypes by their topological properties. Utilizing PRM-derived component classification maps, disease patterns were extracted and quantified to generate imaging surrogates of relevant clinical outcome measures. Although recent studies have evaluated PRM as a quantitative index of disease type (i.e. fSAD and emphysema) and extent (i.e. relative lung volume), the spatial context inherent to this technique is currently underutilized. Evaluation of the spatial distribution and pattern of PRM-derived COPD components may lead to better-informed clinical care through better insight into the heterogeneous clinical subtypes of COPD, with broad application to other obstructive pulmonary diseases (e.g. BOS)^25, 28, 29^.

Different methods have been proposed for quantifying spatial patterns and texture, including fractal analysis^30^, variograms^31^, lacunarity analysis^19^ and Minkowski functionals (MF)^32–34^, of which many have been used to investigate lung diseases. In this study, we have elected to use the Minkowski functionals as open source algorithms are readily available, easily implemented and may be applied to an entire object or computed locally to retain spatial information^31, 33^. As a sensitive measure of dispersed versus clustered disease, we executed these functions (i.e. S, B, χ and α) on PRM classification maps, revealing unique spatial patterns of emphysematous and non-emphysematous air trapping, an indirect measure of small airways disease, as indicators of meaningful clinical measures. By incorporating phenotypic information obtained by PRM with topological analyses, we are able to more fully evaluate information within paired CT data.

The focus in this study was placed on S^i^ due to its high correlation with clinical measures and its stability between Local and Global evaluations. This measure is indicative of texture, with higher values indicating a more dispersed disease and lower values indicating a patchy pattern. Simulated values of S for random distributions (Supplemental Fig. 2) reveal symmetry around a volume fraction of 0.5, intuitive as a positive contrast vs. negative contrast image. This could obfuscate results that span the entire range of volume fractions, which is clearly possible for local analysis on inspection of subject II in Fig. 2. As performed in our two cases, the Local volume density (V^i^) of the PRM classification map was always considered when interpreting values of Local S^i^ (Figs 4, 5, Supplemental Table 4). Although an in-depth evaluation of this effect is warranted, our results clearly show the potential of this technique at extracting meaningful information from the PRM classification maps (Table 1 and Supplemental Table 3).

Extensive work has been performed for stratification of disease phenotypes through analysis of emphysema patterns. Many of these studies have concluded that dispersed emphysema patterns are indicative of an accelerated decline in lung function^4, 34–36^. Because identification of the non-emphysematous component has only recently been attainable, little is known about the correlation of its topological features to clinical outcomes. Wide variations in PRM-derived fSAD distributions are known to exist from qualitative observations, and our results show that fSAD topology, through Local S^fSAD^, is significantly correlated with all clinical measures assessed in this study and may provide complementary insight into the disease than what is attainable through disease extent alone (i.e. % PRM^fSAD^). In fact, based on our findings we postulate that the metric Local S^i^ is capturing varying states of SAD during progression (see Fig. 3). At the early onset of SAD, local parenchyma in the vicinity of affected terminal airways will demonstrate a drop in tissue density (HU on CT scans) from air trapping. This will result in a dispersed pattern in PRM^fSAD^ near the diseased region. As the local disease progresses it may spread throughout the lungs resulting in spirometric decline. Yet within the vicinity of the original onset, the disease may also progress from a dispersed pattern to one that is more concentrated (i.e. clustered, see Fig. 5). It is this critical point that may indicate the onset of emphysema, which would be considered a local end-stage disease.

An important feature of our approach is the retention of spatial information from the original PRM classification maps, which is only attainable through the Local topological analysis. This method is not trivial as it is computationally heavy, requiring long processing times. To reduce our computation times while maintaining sufficient spatial information, local determination of topologic indices was performed using a gridded analysis where our moving window overlaps the subsequent window. Our motivation is to provide each window with sufficient local image information to adequately describe the local metric behavior. However, results are affected by the choice of such parameters as grid spacing and kernel size and shape (Supplemental Methods). Clearly the sensitivity of each topological parameter varies to the process of Local analysis. Regression analyses of Local and Global S to clinical outcomes provided similar findings irrespective of PRM phenotype, i.e. emphysema and fSAD (Table 1 and Supplemental Table 3). In stark contrast, measures χ and α demonstrated mixed results for Local and Global analyses of PRM^fSAD^ classification map, where weak and strong correlations were observed for Local and Global analysis, respectively. Only through local analysis of the topological features were we able to select *ex vivo* core samples based on topological features. From a single case, microCT analysis of these cores allowed us to confirm the extent of disease (i.e. relative volume of PRM^fSAD^) in these regions as well as relate fSAD patterns (i.e. topology of PRM^fSAD^) with physical tissue properties (Fig. 4 and Supplemental Table 4). In addition, we demonstrate the application of our local topological feature metric to monitor local disease coalescence in progressive COPD (Fig. 5). The results presented here provide rationale for further validation studies necessary to statistically correlate *in vivo* PRM topological features to physical phenomena and longitudinal assessment of disease progression.

The following study has limitations that require additional attention. The current study evaluated a methodology that extracted feature patterns from PRM classification maps generated from high-resolution CT data. Our study was fortunate to have access to CT data from a well-controlled multi-center observation COPD trial. Nevertheless, different reconstruction kernels and scanner systems are known to result in variations in HU values. These variations affect the PRM classification maps and resulting topology calculations^37^. Consistent use of reconstruction kernels and scanner is therefore preferred for any prospective analysis of this type. In addition, image resolution is critical for topological comparisons, as lower resolution will intrinsically appear more clustered, biasing the feature patterns in the CT image. Minimal variation in image resolution was found between data sets for this study. Nevertheless, care was taken in accounting for image noise, as well as registration errors, while assessing our metrics^37^. Image noise on typical CT images acquired for this study had a magnitude on the order of 100HU, which could easily translate to the misclassification of voxels and may affect our topological measures by altering the PRM classification maps. Greater noise in the image would be expected to artificially increase dispersion within the PRM classification maps. In the presented analysis, a median filter was applied to each CT image to mitigate this noise effect. The existence of image registration error and its effect on PRM has been the topic of much debate since its original conception, and parameters sensitive to the geometrical distribution of registered data may be particularly biased. A full evaluation of the sensitivity and specificity of our PRM topological approach was not possible. The relatively small number of subjects used in our study limited the power of our statistical analyses. However, it is important to note that these errors would be expected to obfuscate trends rather than create them as seen in our population analysis. Also, many of these concerns are not unique to PRM or our proposed approach, but are a concern to all quantitative CT-based techniques.

We also wish to address the use of individual cases as illustrative examples in order to demonstrate the potential of our new PRM approach. The topological feature indices presented in this study are highly abstract and not easily interpreted. Inclusion of the BOS case (Fig. 4) provides physiological meaning to S^fSAD^ as related to structural differences of the airway and lung parenchyma confirmed by microCT of explanted human lung specimen. Although mechanistically different from COPD, SAD in BOS also is radiographically identified on expiration CT scans as regions of air trapping (low-attenuation regions). In addition, BOS is a SAD-dependent disease allowing us to evaluate our metrics without confounding characteristics, such as emphysema in COPD^28, 38^. Observations from the BOS case study and the 5-yr interval case study (Fig. 5) provide key illustrative examples for physical interpretation of our results. In addition, the finding in our BOS case study, an example of a SAD-dominant disease, provides an important link to our 5-yr interval COPD case finding, in that S^fSAD^ is sensitive to locally-varying structural changes that may indicate a worsening disease state. To confirm these results more extensive studies are required to pathologically validate S^fSAD^ as a measure of SAD and as an indicator of SAD progression. Nevertheless, the findings reported in this study support the assertion that our new PRM analytical approach is able to capture subtle changes in disease progression while maintaining spatial context, which is unattainable using the original PRM concept.

Given the high prevalence and clinical cost of COPD, there is a critical need for further advancements to enable more accurate COPD phenotyping. Beyond COPD, small airway obstruction is a primary manifestation in various other lung diseases, including asthma^39^, obliterative bronchiolitis^40^, and cystic fibrosis^41^. Venegas *et al*.^42^ have recently explored the importance of disease heterogeneity and local interaction between neighboring structures using model simulations of asthma. They have shown that small heterogeneity in ventilation potential produces an imbalance in the system leading to large patched effects, termed self-organized clustering. The ability of the presented method to retain spatial context of local topology could focus clinicians on specific disease-driving lung regions that may be suspect for the onset of emphysema. Our technique may also aid in the targeting of high risk lung regions for more-invasive interventions such as airway brushing, lavage, and biopsy, thus reducing sampling error. Additional work is still required to identify sources of error, test the sensitivity of the technique using large multi-center clinical data, evaluate longitudinal changes in disease pattern and validation using techniques such micro-CT. We introduced in this study an approach for extracting topological readouts from PRM classification maps for characterization of COPD phenotypes. Our method, which has revealed that fSAD pattern, as measured by S^fSAD^, is a key characteristic for assessing disease severity and is a promising next step in providing physicians with actionable data.

## Methods

### Patients

All methods were carried out in accordance with each participating center’s guidelines and regulations. Informed consent was obtained from all participating subjects. Imaging and clinical data were acquired as part of a multicenter (University Medical Center Utrecht (UMCU) and University Medical Center Groningen (UMCG), registered at clinicaltrial.gov, number NCT00807469 and NCT00850863) cross-sectional study of acute and chronic inflammatory responses by smoking^27^. The University Medical Center Utrecht institutional review board and University Medical Center Groningen institutional review board approved all experimental protocols. COPD patients (GOLD stages 1 to 4) were extensively characterized based on pulmonary function tests (post-bronchodilator FEV_1_ and FEV_1_/FVC), diffusion capacity tests, body mass index (BMI), six minute walking distance (6MWD), exacerbation frequency, the number of prednisolone or antibiotic courses in the past year, the Modified Medical Research Council (MMRC) breathlessness scale, BODE index (BMI, degree of airflow obstruction, dyspnea, and exercise capacity), the St. George’s Respiratory Questionnaire (SGRQ) and low-dose chest CT scanning. Of the 95 COPD participants in the study, 3 did not undergo CT acquisitions and 4 could not be evaluated due to image quality issues, resulting in 88 subjects for evaluation (65 male, 23 female) (Supplemental Table 1).

### CT Image Acquisition and Processing

Whole lung volumetric multi-detector CT acquisition was performed at full inspiration and normal expiration using a standard protocol^27^. Briefly, low-dose CT scans at full inspiration (30 mAs at: 90 kVp for patients weighing less than 50 kg, 120 kVp for patients weighing between 50 and 80 kg, and 140 kVp for those weighing more than 80 kg without dose modulation) and expiration (20 mAs at: 90 kVp for patients weighing less than 80 kg and 120 kVp for body mass greater than 80 kg) were acquired. Data was processed using a filtered back projection reconstruction with B30f kernel. High-resolution CT data were presented in Hounsfield Units (HU) with approximately isotropic voxel spacing of 0.7 mm. Stability of CT measurements for each scanner was monitored monthly using a phantom.

Lung parenchyma and airways were segmented from the thoracic cavity to restrict image registration and analysis to lung parenchyma. The inspiratory CT image was spatially aligned to the expiratory CT image. Segmentation and registration of paired CT data were performed using Lung Density Analysis software currently FDA-approved as a medical imaging device (Imbio, LLC, Minneapolis, MN).

### Parametric Response Maps

Prior to image analysis, both inspiratory and expiratory images were filtered using a 2D median filter on each axial slice with a moving window of 3^2^ voxels in order to mitigate the effect of noise on resulting spatial maps. PRM analysis of inspiratory/expiratory lung CT images was performed as previously described^21^. Briefly, registered image voxels (3D discrete image unit consisting of inspiratory and expiratory attenuation values (i.e. HU)) within the segmented lung volume were classified into one of three classifications by imposing two thresholds: (i) −950 HU on inspiratory CT and (ii) −856 HU on expiratory CT. The classifications have been previously reported to identify healthy lung parenchyma (PRM^Normal^, green; >−950 HU on inspiration and >−856 HU on expiration), functional small airways disease (PRM^fSAD^, yellow; >−950 HU on inspiration and ≤−856 HU on expiration), and emphysema (PRM^Emph^, red; ≤−950 HU on inspiration and ≤−856 HU on expiration). Whole-lung measures from PRM analysis are reported as the relative lung volume for each classification (%PRM^i^, where i is an index for fSAD or Emph determined by PRM). In order to minimize the contribution of blood vessels and airways in the analysis, all voxels with HU values >−500 HU in either scan were omitted.

### Topological Analysis

Topological properties of each PRM classification map were explored as independent indicators of clinical outcome (Fig. 1). These topological properties were defined in this study through the Minkowski measures (local estimates of the Minkowski functionals) associated with 3D distributions: Volume (V, in mm^3^), Surface Area (S, in mm^2^), Mean Breadth (B, in mm), and the Euler-Poincaré statistic (χ)^33^. Additional processing with use of the χ statistic produced a condensed descriptor of clustering, α (Supplemental Methods). A detailed description of these parameters is provided in the supplement (Supplemental Methods and Supplemental Fig. 1). Maps of Minkowski measures (i.e. V, S, B, χ and α) were computed using a moving window of size 21^3^ evaluated on a grid of every 5^th^ voxel. Local values from each parameter were normalized to produce parametric densities, with V, S, and B normalized by the masked local window volume and χ and α were normalized by the masked window voxel count. Minkowski measures were quantified per subject as the mean local normalized value over the entire lung volume for group comparisons and regression. For display purposes (Figs 1 and 2 and Supplemental Fig. 4), we multiplied Minkowski measures (S, B, χ and α) by the local density, V, to highlight regions of substantial disease. Final displayed representations of spatially resolved indices have been linearly interpolated back to original dimensions. In addition, global values for V, S, B, χ and α were calculated for each PRM classification over the entire lung volume (Supplemental Methods). The expected behavior of each metric was evaluated using simulations of random distributions at each relative volume (Supplemental Methods and Supplemental Fig. 2). Parameter V is analogous to relative volumes of PRM classification. As such, this parameter was not included in the study analyses. All image processing were performed using in-house algorithms developed in a technical computing language (MATLAB, The MathWorks Inc., Natick, MA).

### Statistical Analysis

Differences in metrics between GOLD were assessed by ANOVA using a Bonferroni post-hoc test to account for multiple comparisons. Topology (i.e. S^i^, B^i^, χ^i^ and α^i^) and extent (%PRM^i^) of disease were evaluated as independent indicators of various clinical outcomes by multivariate linear or logistic regression analysis with stepwise entry. Regression analysis included age, gender, and body mass index (BMI) to account for known clinical correlations. All statistical computations were performed using a statistical software package (SPSS Software Products). Results were considered statistically significant at the two-sided 5% comparison-wise significance level (P < 0.05).

### Case Study: Bronchiolitis Obliterans Syndrome (BOS)

The local hospital’s ethical committee approved the use of this data (S57752). The case used for this analysis was a double lung transplant recipient diagnosed with BOS as part of a single site retrospective clinical study and has been used in a prior study^29^. The subject received azithromycin treatment for BOS but was found to be non-responsive and received a subsequent whole lung transplant surgery allowing for *ex vivo* analysis of the resected lung tissue.

Whole-lung serial paired CT scans were acquired prior to final transplantation. CT scans were obtained at full inspiration (TLC) and relaxed expiration (functional residual capacity) on Siemens Somatom scanner and reconstructed using a b60 or b70 reconstruction kernel. Slices were reconstructed to a thickness of 1.25 mm and acquired volumetrically over the thoracic cavity. Following transplantation, the lung explant was cannulated, inflated near TLC (30 cm of water pressure) and frozen solid in the fumes of liquid nitrogen at −10 cm water pressure and kept at −80 °C. Subsequently, the lungs were cut in frozen condition in 2 cm slices and cores of 1.4 cm diameter were extracted using a cork bore. Frozen cores were subsequently scanned in frozen state using a Skyscan 1172 microCT scanner at a resolution of 10 µm (40 kV, 226 mA) (Skyscan 1172, Brüker microCT, Kontich, Belgium). Reconstruction of scans was done using Nrecon software. Measurements of tissue fraction, surface density, number of obliterations, core volume, and the number of terminal bronchioles were extracted from each frozen core micro-CT in order to correlate with *in vivo* results. To evaluate lung regions with different topographical features, pre-transplant paired CT scans were spatially aligned to photographic images of the uncored and cored explant sections. Detailed methodology of the topological PRM analysis of individual cores is provided in the Supplemental Methods.

### Case Study: COPD Subject with 5-yr Follow-Up

The subject used for this case study was accrued as part of the NIH-funded COPDGene trial. The University of Michigan Institutional Review Board approved the COPDGene research protocol, where all participants provided written informed consent. As part of the COPDGene accrual, individuals had no history of any active lung disease other than COPD as defined by the Global Initiative for Chronic Obstructive Lung Disease criteria. Spirometry was performed using the EasyOneTM spirometry system (ndd Medical Technologies Inc., Zurich, Switzerland) before and after the administration of a short-acting bronchodilator (albuterol). All spirometry tests underwent quality control using both an automated system and manual review. Whole-lung volumetric multidetector CT acquisition was performed at full inspiration and normal expiration using standardized previously published protocol^43^.

## Electronic supplementary material

Supplemental Information

## References

[1] Adeloye D (2015). Global and regional estimates of COPD prevalence: Systematic review and meta-analysis. J Glob Health.

[2] Mannino DM, Buist AS (2007). Global burden of COPD: risk factors, prevalence, and future trends. Lancet.

[3] Caramori G, Kirkham P, Barczyk A, Di Stefano A, Adcock I (2015). Molecular pathogenesis of cigarette smoking-induced stable COPD. Ann N Y Acad Sci.

[4] Barker BL, Brightling CE (2013). Phenotyping the heterogeneity of chronic obstructive pulmonary disease. Clin Sci (Lond).

[5] Pike, D. *et al*. Regional Heterogeneity of Chronic Obstructive Pulmonary Disease Phenotypes: Pulmonary He Magnetic Resonance Imaging and Computed Tomography. *COPD* 1–9 (2016). 10.3109/15412555.2015.1123682 26788765

[6] Han MK (2010). Chronic obstructive pulmonary disease phenotypes: the future of COPD. Am J Respir Crit Care Med.

[7] Agusti A, Vestbo J (2011). Current controversies and future perspectives in chronic obstructive pulmonary disease. Am J Respir Crit Care Med.

[8] Freeman CM (2015). Design of a multi-center immunophenotyping analysis of peripheral blood, sputum and bronchoalveolar lavage fluid in the Subpopulations and Intermediate Outcome Measures in COPD Study (SPIROMICS). J Transl Med.

[9] Capaldi, D.P. *et al*. Pulmonary Imaging Biomarkers of Gas Trapping and Emphysema in COPD: He MR Imaging and CT Parametric Response Maps. *Radiology* 151484 (2016). 10.1148/radiol.2015151484 26744928

[10] Lynch DA, Al-Qaisi MA (2013). Quantitative computed tomography in chronic obstructive pulmonary disease. J Thorac Imaging.

[11] Stolk J (2001). Repeatability of lung density measurements with low-dose computed tomography in subjects with alpha-1-antitrypsin deficiency-associated emphysema. Invest Radiol.

[12] Hoffman EA, Simon BA, McLennan G (2006). State of the Art. A structural and functional assessment of the lung via multidetector-row computed tomography: phenotyping chronic obstructive pulmonary disease. Proc Am Thorac Soc.

[13] Jain N (2005). Quantitative computed tomography detects peripheral airway disease in asthmatic children. Pediatr Pulmonol.

[14] Dijkstra AE (2013). Low-dose CT measurements of airway dimensions and emphysema associated with airflow limitation in heavy smokers: a cross sectional study. Respir Res.

[15] Kurugol S, Washko GR, Estepar RS (2014). Ranking and Classification of Monotonic Emphysema Patterns with a Multi-Class Hierarchical Approach. Proc IEEE Int Symp Biomed Imaging.

[16] Lynch DA (2015). CT-Definable Subtypes of Chronic Obstructive Pulmonary Disease: A Statement of the Fleischner Society. Radiology.

[17] Doel T, Gavaghan DJ, Grau V (2015). Review of automatic pulmonary lobe segmentation methods from CT. Comput Med Imaging Graph.

[18] van Rikxoort EM, van Ginneken B (2013). Automated segmentation of pulmonary structures in thoracic computed tomography scans: a review. Phys Med Biol.

[19] Diaz S (2009). Progression of emphysema in a 12-month hyperpolarized 3He-MRI study: lacunarity analysis provided a more sensitive measure than standard ADC analysis. Acad Radiol.

[20] Mohamed Hoesein FA (2012). Computed tomography-quantified emphysema distribution is associated with lung function decline. Eur Respir J.

[21] Galban CJ (2012). Computed tomography-based biomarker provides unique signature for diagnosis of COPD phenotypes and disease progression. Nat Med.

[22] Boes JL (2015). Parametric response mapping monitors temporal changes on lung CT scans in the subpopulations and intermediate outcome measures in COPD Study (SPIROMICS). Acad Radiol.

[23] Hogg JC (2004). The nature of small-airway obstruction in chronic obstructive pulmonary disease. N Engl J Med.

[24] Bhatt, S. P. *et al*. Association Between Functional Small Airways Disease and FEV Decline in COPD. *Am J Respir Crit Care Med* (2016). 10.1164/rccm.201511-2219OC PMC5003216 26808615

[25] McDonough JE (2011). Small-airway obstruction and emphysema in chronic obstructive pulmonary disease. N Engl J Med.

[26] Stewart JI, Criner GJ (2013). The small airways in chronic obstructive pulmonary disease: pathology and effects on disease progression and survival. Curr Opin Pulm Med.

[27] Loi, A. T. L. *et al*. Acute and chronic inflammatory responses induced by smoking in individuals susceptible and non-susceptible to development of COPD: from specific disease phenotyping towards novel therapy. Protocol of a cross-sectional study. *Bmj Open* **3** (2013). 10.1136/bmjopen-2012-002178 PMC3586075 23377993

[28] Galban CJ (2014). Parametric response mapping as an indicator of bronchiolitis obliterans syndrome after hematopoietic stem cell transplantation. Biol Blood Marrow Transplant.

[29] Verleden SE (2016). Parametric Response Mapping of Bronchiolitis Obliterans Syndrome Progression After Lung Transplantation. Am J Transplant.

[30] Uppaluri R, Mitsa T, Sonka M, Hoffman EA, McLennan G (1997). Quantification of pulmonary emphysema from lung computed tomography images. Am J Respir Crit Care Med.

[31] Jacob RE, Carson JP (2014). Automated measurement of heterogeneity in CT images of healthy and diseased rat lungs using variogram analysis of an octree decomposition. BMC Med Imaging.

[32] Larkin TJ (2014). Analysis of image heterogeneity using 2D Minkowski functionals detects tumor responses to treatment. Magn Reson Med.

[33] Legland D, Kieu K, Devaux M (2007). Computation of Minkowski Measures on 2D and 3D Binary Images. Image Anal Stereol.

[34] Charemza, M. T., Bhalerao, E., Parr, A. D. Integral Geometry Descriptors for Characterizing Emphysema and Lung Fibrosis in HRCT Images. In *First International Workshop on Pulmonary Image Analysis* 155–164 (New York, 2008).

[35] Boehm HF (2008). Automated classification of normal and pathologic pulmonary tissue by topological texture features extracted from multi-detector CT in 3D. Eur Radiol.

[36] Mohamed Hoesein FA (2011). CT-quantified emphysema in male heavy smokers: association with lung function decline. Thorax.

[37] Boes JL (2015). The Impact of Sources of Variability on Parametric Response Mapping of Lung CT Scans. Tomography.

[38] Belloli, E. A. *et al*. Parametric Response Mapping as an Imaging Biomarker in Lung Transplant Recipients. *Am J Respir Crit Care Med* (2016). 10.1164/rccm.201604-0732OC PMC5387704 27779421

[39] Lutchen KR (2001). Airway constriction pattern is a central component of asthma severity: the role of deep inspirations. Am J Respir Crit Care Med.

[40] Lynch JP (2012). Obliterative (constrictive) bronchiolitis. Semin Respir Crit Care Med.

[41] Balfour-Lynn IM, Elborn JS (2002). “CF asthma”: what is it and what do we do about it?. Thorax.

[42] Venegas JG (2005). Self-organized patchiness in asthma as a prelude to catastrophic shifts. Nature.

[43] Regan EA (2010). Genetic epidemiology of COPD (COPDGene) study design. COPD.

