# Supplementary material for: CT-Based Local Distribution Metric Improves Characterization of COPD
Source: Sci Rep. 2017 Jun 7;7:2999. doi: 10.1038/s41598-017-02871-1 (PMC5462827; doi:10.1038/s41598-017-02871-1)
Supplement: Supplementary file 1 — Supplemental Information [file 41598_2017_2871_MOESM1_ESM.pdf]

CT-Based Local Distribution Metric Improves Characterization of COPD

Benjamin A. Hoff, Esther Pompe, Stefanie Galbán, Dirkje S. Postma, Jan-Willem J. Lammers, Nick H. T. ten Hacken, Leo Koenderman, Timothy D. Johnson, Stijn E Verleden, Pim A. de Jong, Firdaus A. A. Mohamed Hoesein, Maarten van den Berge, Brian D. Ross, Craig J. Galbán

Supplemental Information Guide

Supplementary Item	Title of Caption
Supplementary Figure 1	Minkowski Functionals
Supplementary Figure 2	Simulations of %PRM vs Topology Metrics
Supplementary Figure 3	Local vs Global Topological Measures
Supplementary Figure 4	Representative Cases with Spatially Resolved Topological Measures
Supplementary Figure 5	%PRM vs Topology
Supplementary Figure 6	Trends in Topological Measures with GOLD
Supplementary Figure 7	Case Study: Monitoring Progression of Obstructive Disease
Supplementary Table 1	Patient Characteristics
Supplementary Table 2	Topological Metric Comparisons between GOLD
Supplementary Table 3	Multivariate Regression
Supplementary Table 4	Comparison of Topological Metrics to MicroCT Measurements
Supplementary Methods	Calculation of Morphological Indices
Supplementary Methods	Simulations
Supplementary Methods	Case Study: PRM and Topological Feature Analysis of Lung Cores

Supplemental Figure 1

Minkowski Functionals

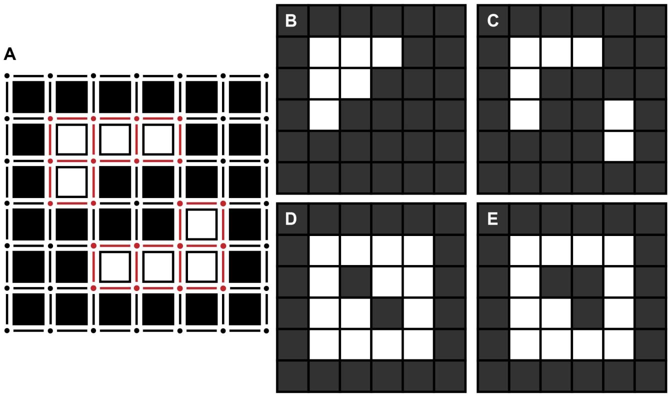

Supplemental Figure 1: Illustrative examples of 2D patterns with corresponding Minkowski analysis. (A) Binarized 2D images are decomposed into counts of squares (n_s), edges (n_e), and vertices (n_v) (for 3D: cubes (n_c), faces (n_f), edges (n_e), and vertices (n_v)). Minkowski functionals are then calculated to determine area (A), perimeter (U), and genus (χ) (for 3D: volume (V), surface area (S), mean breadth (B), and Euler-Poincaré characteristic (χ)). Further examples show Minkowski functionals of: (B) A = 6, U = 12, χ = 1; (C) A = 7, U = 18, χ = 2; (D) A = 14, U = 24, χ = -1 (E) A = 13, U = 24, χ = 0.

Minkowski functionals, calculated over the entire lung volume (Global) and sub-volumes calculated on a moving window (Local), were used in this study to describe the topology of disease distribution. Metrics were normalized by masked window volumes (for V, S, and B) or voxel counts (for χ and α) in order to provide density approximations that are comparable and unaffected by mask edges. The representative 2D patterns in **Supplemental Figure 1** use a simplified counting scheme without adjustments for grid resampling in order to easily illustrate how these measures are calculated and provide simplified meaning. Actual calculations were performed on 3D volumes with Minkowski measure estimates using the equations derived by Legland et al. [1].

Supplemental Figure 2

Simulations of %PRM vs Topology Metrics

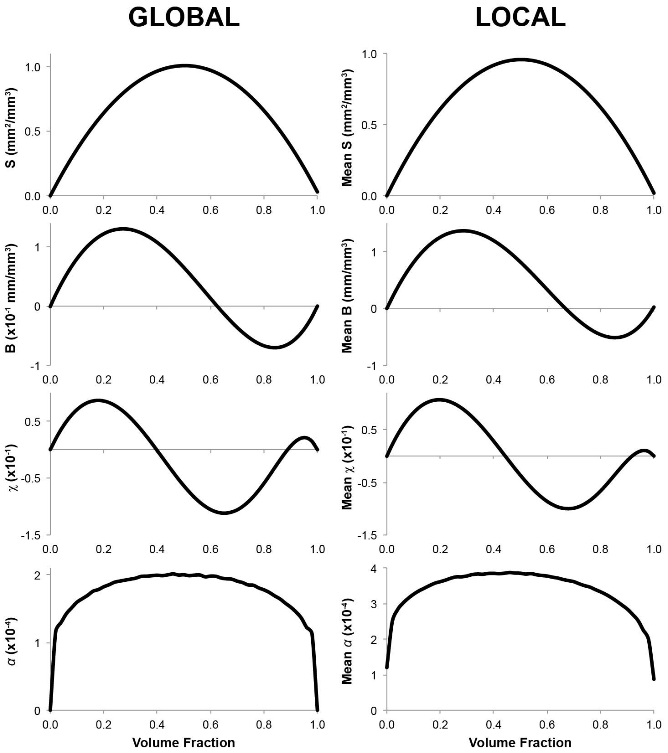

Supplemental Figure 2. Simulated results show typical behavior of global (left) and local (right) measures against volume fractions of the binary map (e.g. PRM^{fSAD} classification map). Local measures are presented as the mean value over the masked volume.

Simulations were performed to demonstrate typical Minkowski measure behavior. Similar trends were seen between Global and mean Local measures, with the greatest difference in the magnitude of the α values (mean Local $\boldsymbol{\alpha}$ = ~2x Global $\boldsymbol{\alpha}$). All topological measures tend to approach 0 at the limits of volume fraction (i.e. 0 and 1). Values of S and α tend to peak at a volume fraction around 0.5, while B and χ show a oscillating trend about 0. Parabolic trends in S reflect the similarity between a binary pattern and its negative. For example, a binary map consisting of only zeros and ones have no surface area (S=0). Similarly, the behavior of χ shows many small connected regions (positive counts) at low relative volumes that shift to a greater number of tunnels (negative counts) than connected bodies at greater volume fractions. These results are intended to aid in the translation of results into physically meaningful interpretations.

The ability of multiple volume fractions to translate to the same topological measure could be an important factor in the use of individual measures to provide meaningful results. Minkowski measures on fSAD for this study can clearly span the entire range of volume fractions within a single data set, as evidenced by Subject II in **Figure 2**. In this patient, clustered fSAD (at the top of the bottom right lobe) appears as a ring of high S^{fSAD}, with regions of high V^{fSAD} in the center and lower

V^{fSAD} on the outside having equivalent S^{fSAD} measures. Since subject comparisons were made on the mean local measures, this behavior may obfuscate results. Nonetheless, S^{i} was found to have significant independent correlation with clinical observations. Future work will explore sources of error and optimal interpretation of the Minkowski measures.

Supplemental Figure 3

Local vs Global Topological Measures

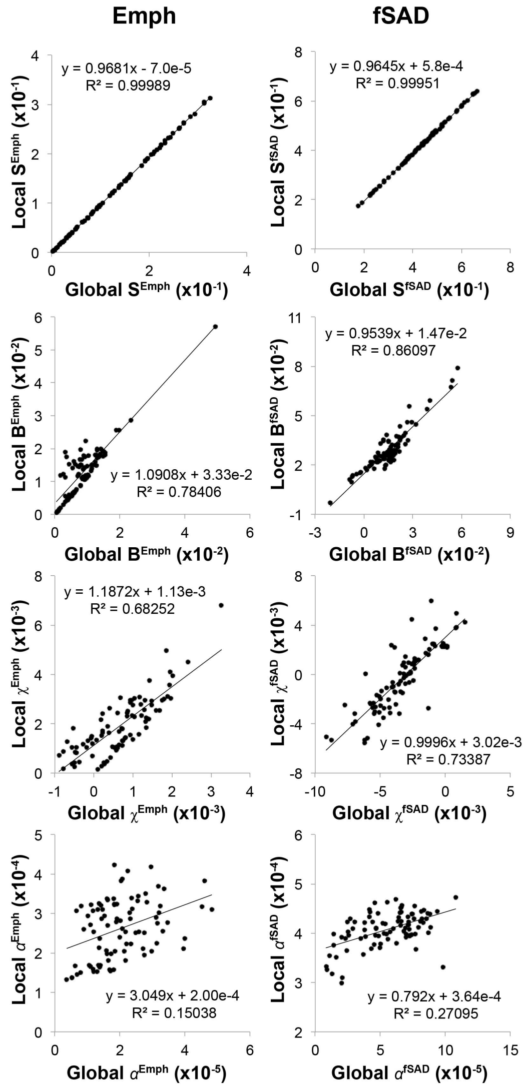

Supplemental Figure 3: Scatter plots comparing local measures against their respective global measures. Linear fits were performed to determine agreement between measurements. Linear equation and R^2 are provided.

Global measures were plotted against respective mean of local measures to show agreement between methods and qualify global evaluation as a rough readout. Values of S displayed a high degree of agreement between local and global analysis, with R^2 values >0.999 for both $\%PRM^{Emph}$ and $\%PRM^{fSAD}$. Higher order measures were found to have lower correlation between measurements, suggesting more sensitivity of metrics to windowing effects. Measures of α were found to have very poor correlation, most likely due to lack of sufficient image information, so care must be taken in the interpretation of such local results.

Supplemental Figure 4

Representative Cases with Spatially Resolved Topological Measures

I : Diffuse II : Aggregated

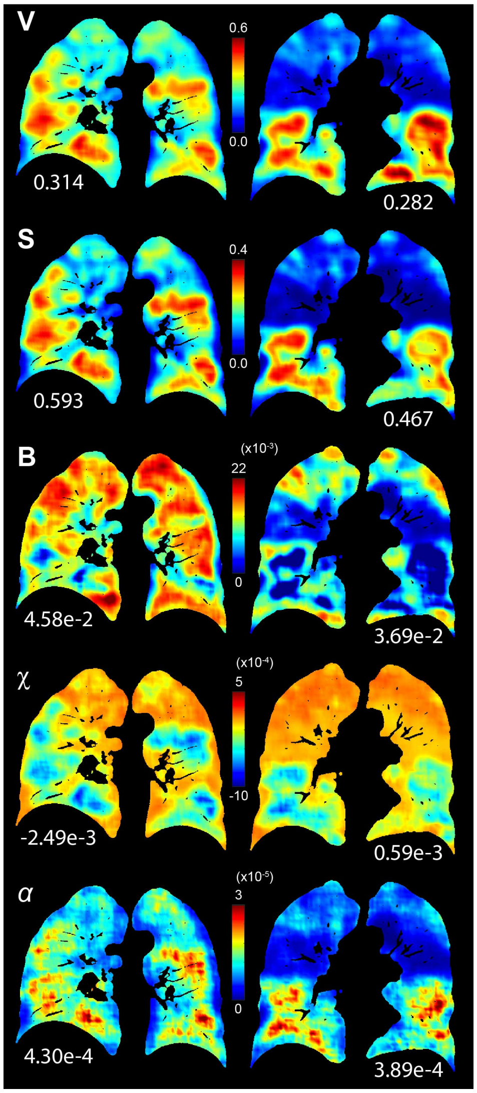

Supplemental Figure 4: Local measures [multiplied by local density (V)] of diffuse vs. aggregated fSAD demonstrate the potential for local topology to highlight subtypes within GOLD status. Mean local values are indicated for each case for comparison.

Spatial maps (**Supplemental Figure 4**) of all topological measures were generated for the two representative cases introduced in **Figure 2**. Clear differences in the topological pattern of each measure are apparent between subjects, revealing signatures of disease distribution that may be clinically relevant.

Supplemental Figure 5

%PRM vs Topology

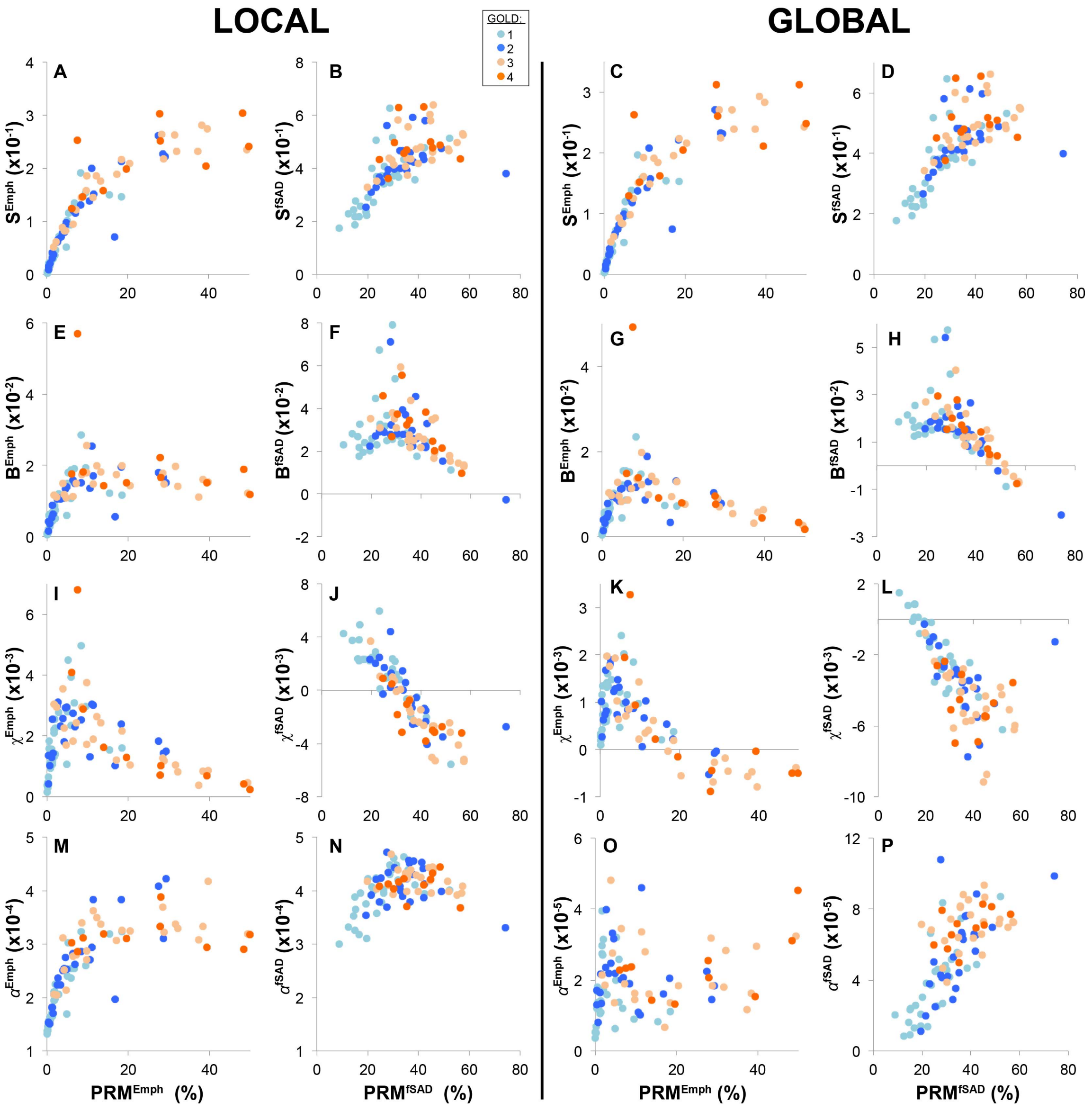

Supplemental Figure 5: Comparative plots between %PRM (emphysema, first and third column, or fSAD, second and fourth column) and their respective topological measures. Individuals are color coded by GOLD status.

Relationships between %PRM and respective topological measures are shown in **Supplemental Figure 5**. A color legend identifies the GOLD status of each subject. $\%PRM^{Emph}$ in this study spanned the range under approximately 50%, while $\%PRM^{fSAD}$ spanned a higher range (up to ~74%). All measures followed similar trends as seen in the simulated results, with deviations in topological measure at similar volume fractions indicative of topological disease signatures. The global measures of α^{Emph} demonstrated the largest spread in the data.

Supplemental Figure 6

Trends in Topological Measures with GOLD

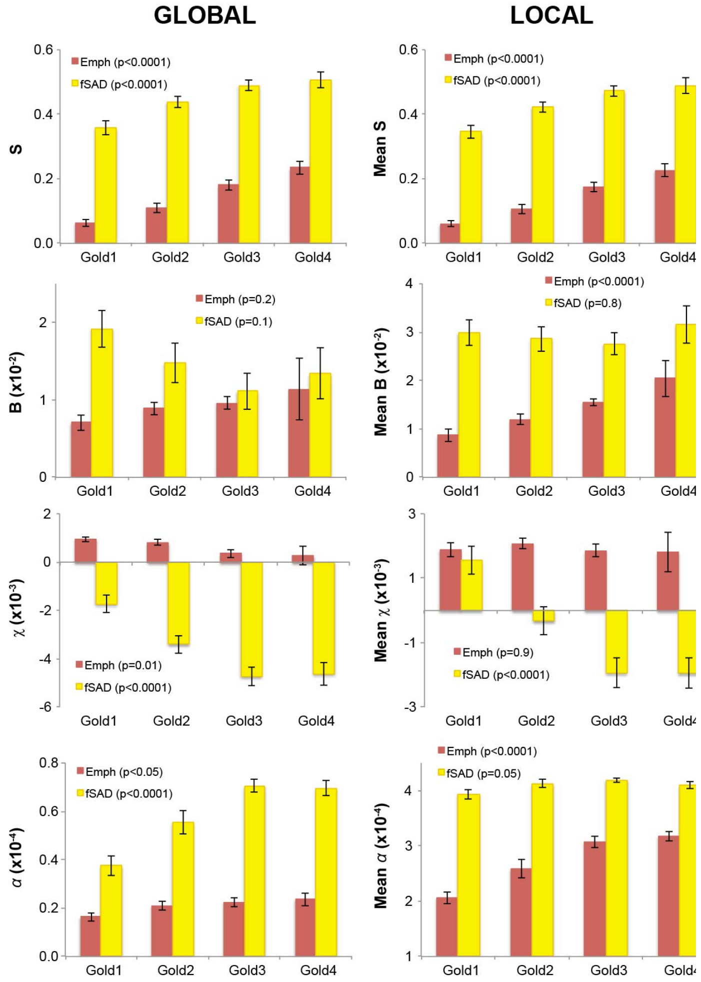

Supplemental Figure 6: Plots show mean measures for each GOLD status (group variations are represented as standard error of the mean; global on the left, local on the right). P-values for each plot are provided. Between-groups comparison were performed using an one-way ANOVA.

Mean values in topological measures, shown in **Supplemental Figure 6**, reveal clear trends with lung function (i.e. GOLD). S^i, both local and global for Emph and fSAD, was found to increase with increasing GOLD status. B^i was the weakest indicator of lung function, and did not trend with GOLD status, with the exception of local analysis on PRM^{Emph}. Measures of χ^{fSAD} decreased with GOLD status.

Supplemental Table 1

Parameter	GOLD 1	GOLD 2	GOLD 3	GOLD 4
Number	29	24	24	11
Gender (M/F)	23/6	20/4	17/7	5/6
Age (years)	63 (7)	64 (6)	61 (7)	62 (5)
BMI (kg/cm^2)	27 (5)	26 (3)	24 (4)	25 (6)
Pack-years	41 (18)	33 (9)	43 (18)	38 (22)
FEV1 (% predicted)	95 (9)	64 (10)	41 (6)	29 (9)
FEV1/FVC	0.63 (0.06)	0.50 (0.10)	0.37 (0.06)	0.31 (0.07)
SGQR	20 (12)	29 (13)	38 (13)	53 (14)
6-min. Walk	527 (73)	543 (78)	457 (83)	358 (92)
MMRC	1.3 (0.7)	1.8 (0.9)	2.5 (1.2)	3.6 (1.4)
BODE	0.1 (0.4)	0.8 (1.0)	3.0 (1.5)	5.0 (1.7)
$\%PRM^{Emph}$	4 (5)	9 (9)	18 (14)	27 (18)
$\%PRM^{fSAD}$	25 (10)	35 (11)	40 (10)	38 (10)
$\%PRM^{Norm}$	70 (13)	55 (15)	40 (13)	33 (15)

* Values are displayed as mean (standard deviation)

Supplemental Table 2

Topological Metric Comparisons between GOLD

			Global			Local		
			GOLD1	GOLD2	GOLD3	GOLD1	GOLD2	GOLD3
Emph	S	GOLD2	0.0926			0.0951		
		GOLD3	0.0000	0.0036		0.0000	0.0032	
		GOLD4	0.0000	0.0000	0.1955	0.0000	0.0000	0.1954
	B	GOLD2	1.0000			0.4919		
		GOLD3	0.8937	1.0000		0.0027	0.4461	
		GOLD4	0.3250	1.0000	1.0000	0.0000	0.0061	0.3120
	χ	GOLD2	1.0000			1.0000		
		GOLD3	0.0439	0.3054		1.0000	1.0000	
		GOLD4	0.1004	0.3973	1.0000	1.0000	1.0000	1.0000
	α	GOLD2	0.4189			0.0151		
		GOLD3	0.1072	1.0000		0.0000	0.0400	
		GOLD4	0.1620	1.0000	1.0000	0.0000	0.0545	1.0000
fSAD	S	GOLD2	0.0183			0.0211		
		GOLD3	0.0000	0.3788		0.0000	0.4187	
		GOLD4	0.0002	0.3045	1.0000	0.0002	0.3314	1.0000
	B	GOLD2	1.0000			1.0000		
		GOLD3	0.1167	1.0000		1.0000	1.0000	
		GOLD4	1.0000	1.0000	1.0000	1.0000	1.0000	1.0000
	χ	GOLD2	0.0108			0.0142		
		GOLD3	0.0000	0.0973		0.0000	0.0743	
		GOLD4	0.0002	0.4654	1.0000	0.0001	0.2639	1.0000
	α	GOLD2	0.0073			0.2798		
		GOLD3	0.0000	0.0530		0.0517	1.0000	
		GOLD4	0.0001	0.2917	1.0000	1.0000	1.0000	1.0000

Comparison of mean topological metrics between GOLD status groups was performed using a One-Way ANOVA using Bonferroni post-hoc to control for multiple comparisons. P-values are shown in **Supplemental Table 1**, with significance determined at the 95% level (highlighted in orange). Overall between-group significance is also shown for each topological feature of PRM^{Emph} and PRM^{fSAD} in the plot legends of **Supplemental Figure 4**. Global evaluation of PRM^{Emph} did not reveal significant between-group differences, with the exception of S^{Emph}. Local evaluation of PRM^{Emph} revealed significant trends between groups except for χ^{Emph}. PRM^{fSAD} trends between groups were found significant in S^{fSAD} and χ^{fSAD} for both global and local analysis, while only global α^{fSAD} was significantly different between groups.

Supplemental Table 3

Multivariate Regression

			PRM%	S		PRM%	B		PRM%	χ		PRM%	α	
Mean of Local	fSAD	FEV_1	-	<0.0001	d	<0.0001	0.0048	d	-	<0.0001	b	<0.0001	-	b
		FEV_1/FVC	0.0114	0.0131	d	<0.0001	-		-	<0.0001		<0.0001	-	
		SGRQ total score	-	0.0006		-	-		-	0.0196		-	-	
		6-min walk distance	0.0091	<0.0001		0.0160	<0.0001		-	-	c	-	0.0227	c
		MMRC dyspnea scale score[a]	-	<0.0001		-	-		-	-		-	-	
		BODE score[a]	-	<0.0001		0.0151	-		-	0.0018		0.0151	-	
	Emph	FEV_1	-	<0.0001	b	<0.0001	0.0007	b	<0.0001	0.0042	b	0.0025	0.0003	b
		FEV_1/FVC	-	<0.0001		<0.0001	<0.0001		<0.0001	0.0024		0.0002	<0.0001	
		SGRQ total score	<0.0001	-	d	<0.0001	-	d	<0.0001	-	d	<0.0001	-	d
		6-min walk distance	<0.0001	-	c,d	<0.0001	-	c,d	<0.0001	-	c,d	<0.0001	-	c,d
		MMRC dyspnea scale score[a]	-	<0.0001		<0.0001	-	d	<0.0001	-	d	<0.0001	-	d
		BODE score[a]	-	<0.0001		<0.0001	0.2581		<0.0001	-		<0.0001	-	
Global	fSAD	FEV_1	-	<0.0001	d	<0.0001	-		-	<0.0001	d	-	<0.0001	d
		FEV_1/FVC	0.0144	0.0107	d	<0.0001	-		-	<0.0001	d	0.0099	0.0047	d
		SGRQ total score	-	0.0005		-	-		-	0.0011		-	<0.0001	
		6-min walk distance	0.0070	<0.0001		-	-	c	0.0109	<0.0001		-	<0.0001	
		MMRC dyspnea scale score[a]	-	0.0339		-	-		-	0.0401		-	0.0109	
		BODE score[a]	-	0.0002		0.0151	-		-	0.0001		0.0151	-	
	Emph	FEV_1	-	<0.0001	b	<0.0001	0.0016	b	<0.0001	-	b	<0.0001	0.0435	b
		FEV_1/FVC	-	<0.0001		<0.0001	0.0001		<0.0001	-		<0.0001	-	
		SGRQ total score	<0.0001	-	d	<0.0001	-	d	<0.0001	-	d	<0.0001	-	d
		6-min walk distance	<0.0001	-	c,d	<0.0001	-	c,d	<0.0001	-	c,d	<0.0001	-	c,d
		MMRC dyspnea scale score[a]	-	<0.0001		<0.0001	-	d	<0.0001	-	d	<0.0001	-	d
		BODE score[a]	-	<0.0001		<0.0001	-		<0.0001	-		<0.0001	-	

Note: Presented are the *P values* generated from stepwise regression models including individual topological indices and their respective PRM value (and age, gender, and body mass index (BMI)). FEV_1 = forced expiratory volume in one second (% predicted); FVC = forced vital capacity; SGRQ = St. George's Respiratory Questionnaire; MMRC = Modified Medical Research Council; BODE = body mass index, degree of airflow obstruction, dyspnea, and exercise capacity. [a] denotes use of a logistic instead of linear regression, [b] denotes age was significant, [c] denotes gender was significant, and [d] denotes BMI was significant. Parameters not included in the model due to lack of significant effects are marked with a dash.

Supplemental Table 4

Comparison of Topological Metrics to MicroCT Measurements

	A	B
**%PRM^{fSAD}**	76.1	68.4
**Local V^{fSAD}**	0.572	0.589
**Local S^{fSAD}**	0.244	0.381
**Local B^{fSAD}**	-0.0030	-0.0035
**Local χ^{fSAD}**	-0.0033	-0.0071
**Tissue (%)**	23.4	28.0
**Surface Density**	0.011	0.012
**Obliterations**	2	0
**Core Volume**	2.743	2.769
**Terminal Bronchioles**	8	6

Supplementary Methods

Calculation of Morphological Indices

Topological metrics in this study were defined using the 3D Minkowski measures defined in Legland et al. [1], with 3-directionality and 6-connectivity. Minkowski measure estimates ignore intersections between the structure and the window border in order to consider the image as a representative window of a larger structure. A few illustrative patterns in 2D are shown in **Supplemental Figure 1** to further illustrate a counting procedure for determination of 2D Minkowski functionals: area (A), perimeter (U), and the 2D Euler-Poincaré characteristic (χ). In general, for the same volume fraction of binary 1's, one would expect a greater S to indicate a more diffuse pattern while a lower value would indicate a more aggregated pattern. Similarly, a greater value of B would indicate that connected regions span a greater length than lower values of B. The value of χ is roughly indicative of the number of connected regions plus the number of completely enclosed holes minus the number of tunnels. This is apparent in the representative images in **Supplementary Figure 4**, where negative values (toward blue) indicate fewer, likely larger, connected regions of fSAD with many tunnels while positive values (toward red) indicate a sparse pattern with many small connected regions.

Additional post-processing using the χ metric was performed to calculate a condensed descriptor of aggregation (α) [2]. In brief, binary PRM maps were first smoothed using a Gaussian filter with standard deviation of 2 voxels in each direction to generate a density map. The smoothing length determines the scale of interest for groupings. χ was then determined at various thresholds (u), between 0 and 1 (101 equally spaced thresholds for the purposes of this study), and averaged using the following equation [2]:

$$\alpha = \sqrt{\int_0^1 (\chi(u) - 1)^2 du}$$

The aggregation value is larger with greater heterogeneity in the binary map. The approximation of the χ measure, therefore α as well, strongly depends on the choice of adjacency system. The use of 6-connectivity for this study was chosen for simplicity, and exploration of higher connectivity systems could prove beneficial, however such analysis was beyond the scope of this preliminary study.

Simulations

We analyzed random distributions at various volume fractions in order to show typical behavior of each Minkowski measures. Random binary distributions were generated inside a spherical mask with matrix size 256^3 and mask radius of 200^3. A total of 10 iterations were performed at each volume fraction, with 51 volume fractions spaced evenly between 0 and 1. Aggregation values were determined using 101 equally spaced thresholds (u) from 0 to 1. Local analysis was performed using the same grid spacing that was used in the clinical analysis. Both local and global analyses were

performed for each randomized distribution, and local comparisons were evaluated as the mean local measure over the masked volume. Plots of topological feature trends against relative volume are displayed in **Supplemental Figure 2** as the mean of measurements. Confidence intervals at the 95% level were determined over the range of volume fractions, however they were too close to the mean to be seen in the plots and are not shown.

Case Study: PRM and Topological Feature Analysis of Lung Cores

Bronchiolitis obliterans syndrome is a chronic lung allograft dysfunction in transplant recipients. It is known to result in air trapping which is detectable by CT, and is characterized by a spirometric decline and histopathalogical observation of obliterative bronchiolitis (OB) [3,4]. In order to correlate *in vivo* analysis results with histopathology, we provide a case study with cored lung analysis to serve as a representative sample and provide rationale for further validation studies.

Topological features of individual cores were determined through spatial alignment of the *in vivo* paired CT scans to a photograph of the explanted cored lung section. The process involves the following 5 steps. First, pre-transplant inspiration CT scan was spatially aligned to the paired expiration CT scans using a deformable registration algorithm as described in the main text. Second, The lung used for the microCT analysis was segmented from the thoracic cavity in the pre-transplant expiration CT scan and spatially aligned to the CT scan of the explanted inflated frozen lung using the same registration algorithm. Third, to register the inflated lung to the uncored section RGB photograph additional post-processing was required. The photograph of the uncored section was converted to gray scale, subsampled by a factor of 10 and a histogram equalization algorithm was applied to improve image contrast. To obtain the 3D PRM^{fSAD} classification map within the core, the image of the uncored lung section was replicated such that a 3D data set was constructed with the same number of slices as the inflated frozen lung 3D CT scan. The explant image on the center slice was then segmented. A similarity transformation, i.e. rotate-translate-isotropic scaling, was performed to spatially align the inflated lung CT scan to the segmented uncored section in the dataset. Fourth, post-processing of the photograph of the uncored lung section and the third step were repeated to register the uncored lung section to the cored lung section. Finally, the transformation matrices from each step were applied to the pre-transplant paired CT scans. PRM classification and resulting topological maps were generated from the paired CT data aligned to the cored section. Mean values for the topological features were calculated for the individual cores. The core volume of interest was determined by contouring the cored volume in the 3D cored lung section dataset. The number of contoured slices was determined by dividing the section thickness by the transformed voxel size (35 slices=20mm section /[0.82 scaling factor*0.7mm slice thickness for inflated lung CT scan]).

References

1. Legland, D., Kieu, K. & Devaux, M. Computation of Minkowski Measures on 2D and 3D Binary Images. *Image Anal Stereol* **26**, 83-92 (2007).
2. Beisbart, C., Buchert, T. & Wagner, H. Morphometry of spatial patterns. *Physica A* **293**, 592-604 (2001).
3. Verleden, S.E.*, et al.* Linking clinical phenotypes of chronic lung allograft dysfunction to changes in lung structure. *Eur Respir J* **46**, 1430-1439 (2015).
4. Verleden, S.E.*, et al.* The site and nature of airway obstruction after lung transplantation. *Am J Respir Crit Care Med* **189**, 292-300 (2014).
